# Paradoxical restoration from complete and persistent atrioventricular block after surgical aortic valve replacement: a case report

**DOI:** 10.1093/ehjcr/ytae549

**Published:** 2024-10-05

**Authors:** Ami Nishihara, Yuta Okabe, Sei Morizumi, Yoshiharu Enomoto, Kentaro Yoshida

**Affiliations:** Department of Cardiology, Ibaraki Prefectural Central Hospital, 6528 Koibuchi, Kasama 309-1793, Japan; Department of Cardiology, Ibaraki Prefectural Central Hospital, 6528 Koibuchi, Kasama 309-1793, Japan; Department of Cardiology, Institute of Medicine, University of Tsukuba, Tsukuba 305-8575, Japan; Department of Cardiovascular Surgery, Ibaraki Prefectural Central Hospital, Kasama 309-1793, Japan; Department of Cardiovascular Surgery, Ibaraki Prefectural Central Hospital, Kasama 309-1793, Japan; Department of Cardiology, Ibaraki Prefectural Central Hospital, 6528 Koibuchi, Kasama 309-1793, Japan; Department of Cardiology, Institute of Medicine, University of Tsukuba, Tsukuba 305-8575, Japan

**Keywords:** Syncope, Complete atrioventricular block, Aortic stenosis, Aortic valve replacement, Case report

## Abstract

**Background:**

One of the most important and relatively frequent complications of aortic valve replacement is atrioventricular block. It typically occurs by direct injury of the infranodal conduction system due to intra-operative manipulation and persists post-operatively, necessitating permanent pacemaker implantation in many cases.

**Case summary:**

A 66-year-old man presented to our hospital after experiencing syncope while walking after drinking. He had experienced two episodes of alcohol-induced syncope several years earlier. His electrocardiogram (ECG) and transthoracic echocardiogram revealed complete atrioventricular block and severe aortic stenosis, respectively. He received a temporary pacemaker on the day of admission and underwent surgical aortic valve replacement on hospital Day 9. The native aortic valve was bicuspid. Unexpectedly, the ECG immediately after aortic valve replacement showed complete restoration of atrioventricular conduction during temporary atrial pacing. The atrioventricular block did not recur, and he was discharged to home on post-operative Day 13.

**Discussion:**

This remarkably rare clinical course, complete restoration from complete and persistent atrioventricular block after surgical aortic valve replacement, can be explained by multifactorial mechanisms: (i) surgical removal of the aortic annulus calcification directly hindering the infranodal conduction system; (ii) relief from the ventricular pressure overload stressing the conduction system within the septum; and (iii) improvement of substantial autonomic dysregulation as manifested by alcohol-sensitive syncope in the present patient, which was a result of unloading of the intraventricular pressure affecting the left ventricular mechanoreceptor.

**Learning points**

- Paradoxical restoration from complete and persistent atrioventricular block after surgical aortic valve replacement presents as an extremely rare clinical course.
- Multifactorial mechanisms, including haemodynamic impairment, mechanical compression of the conduction system by valvular calcification, autonomic imbalance, and hypersensitivity of the stretch-induced mechanoreceptor, may be the causes of this unexpected phenomenon.

## Introduction

Aortic valve replacement (AVR) is the only radical therapy for severe aortic valvular diseases. Because of the anatomical proximity of the aortic valvular apparatus to the infranodal conduction system, atrioventricular (AV) block (AVB) is an important post-operative complication. We report the rare and paradoxical clinical course of a patient with complete and persistent AVB and severe aortic stenosis (AS) who underwent surgical AVR and subsequently fully recovered from AVB.

## Summary figure

**Figure:**
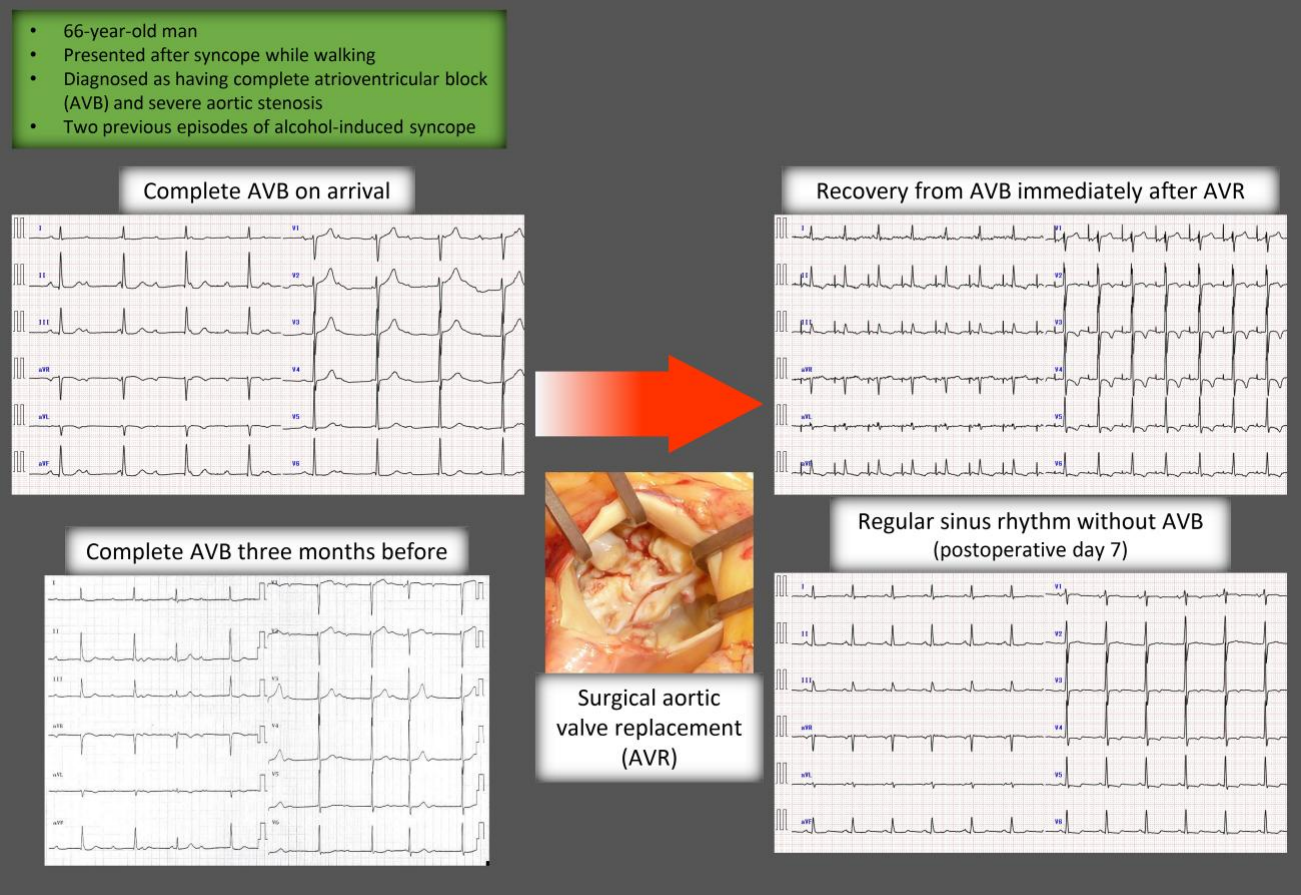


## Case presentation

**Figure 1:**
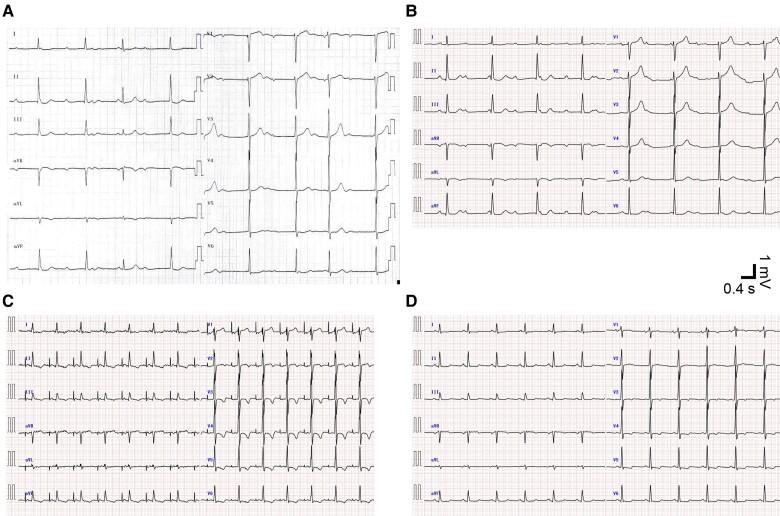
Time series of electrocardiograms. Vertical and horizontal bars denote 1 mV and 0.4 s, respectively. (*A*) Twelve-lead electrocardiogram of complete atrioventricular block 3 months before the present visit shows electrical atrioventricular dissociation. (*B*) Twelve-lead electrocardiogram of complete atrioventricular block at the present visit. (*C*) Twelve-lead electrocardiogram immediately after surgical aortic valve replacement shows spontaneous R waves following temporary atrial pacing. Atrial pacing spike to R-wave duration is 190 ms. (*D*) Twelve-lead electrocardiogram on post-operative Day 7 shows regular sinus rhythm with a PR interval of 154 ms.

A 66-year-old man was transported to our hospital after experiencing syncope and occipital contusion while walking his dog after drinking alcohol. His electrocardiogram (ECG) at his medical check-up 3 months earlier showed complete AVB (*Figure 1A*). As he was asymptomatic and the automatic interpretation of the ECG did not indicate AVB, he underwent elective examination for AVB in another hospital 9 days prior to the present visit, where severe AS (peak velocity: 4.9 m/s) was also revealed. In accordance with the ESC guidelines,^1^ pacemaker implantation was recommended, but he refused for family reasons at that time.

He had a history of hypertension and already experienced two episodes of syncope at 3 and 4 years, respectively, before the present episode, both after drinking. Neither prolongation of his PR interval nor severe valvular disease was evident on medical examinations following these previous episodes.

**Figure 2:**
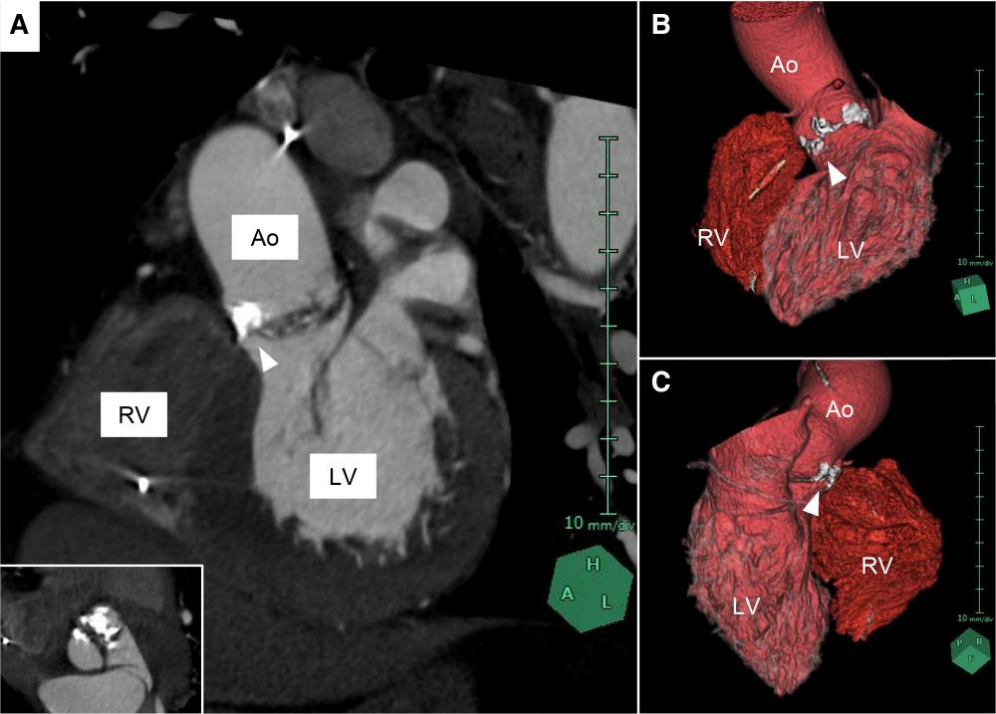
Calcified aortic valve complex on computed tomography. (*A*) Left anterior oblique cross-sectional image reveals heavy aortic valve calcification extending to the interventricular membranous septum. Inset image shows a view parallel to the aortic valve. (*B* and *C*) 3D images reconstructed from computed tomography. White arrowheads indicate calcification attached to aortic valve. Ao, ascending aorta; LV, left ventricle; RV, right ventricle.

The present ECG showed complete AVB with junctional escape beats (*Figure 1B*), similar to his ECG 3 months earlier (*Figure 1A*), suggesting persistent rather than transient AVB. Physical examinations and laboratory studies revealed a systolic ejection murmur and negative for high-sense troponin T and normal electrolyte concentrations, respectively. Transthoracic echocardiography revealed severe AS [peak velocity, 4.4 m/s; mean pressure gradient, 44 mmHg; and aortic valve area, 0.84 cm^2^ (continuity equation)] with normal left ventricular contraction and wall thickness (Supplementary material online, *Videos S1*). Electrocardiogram-synchronized contrast-enhanced computed tomography showed severe aortic valve calcification without significant coronary stenosis (*Figure 2*).

**Figure 3:**
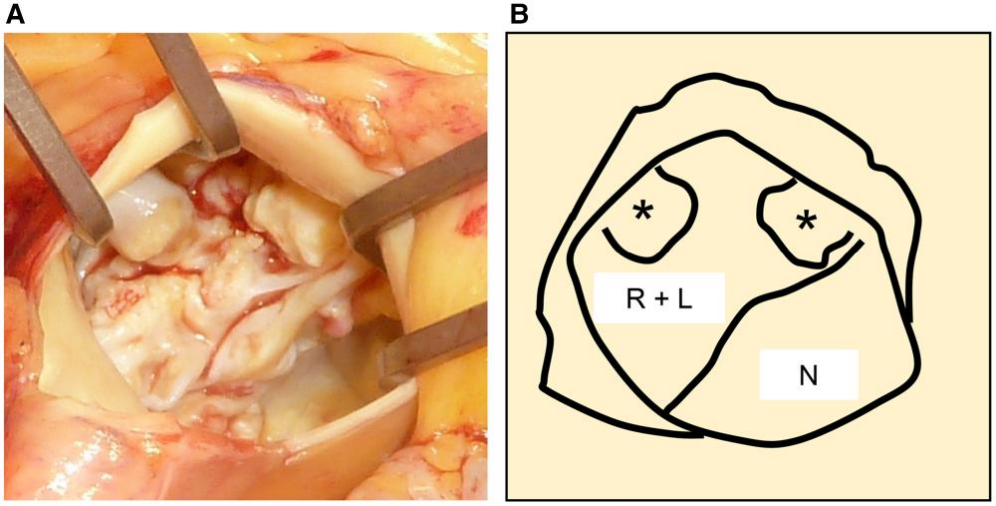
Intra-operative findings of the aortic valve. (*A*) Intra-operative image of the aortic valve from the aortic side. (*B*) Schema of the image. Asterisks indicate heavy calcifications. R + L, fused right and left cusps; N, non-coronary cusp.

On patient admission, we initiated temporary transvenous cardiac pacing for symptomatic advanced AVB with indication of bradycardia-related cardiogenic syncope, in compliance with the guidelines.^1^ As current guidelines do not provide a treatment strategy for AS with complete AVB, we chose surgical AVR first rather than permanent pacemaker implantation. Our reasons were that he was a good candidate for surgical AVR because he was relatively young with minimal comorbidities and to eliminate the risk of implantable electronic device infections resulting from transient bacteremia associated with a surgical procedure.^2^ He underwent surgical AVR on hospital Day 9. The native aortic valve was bicuspid, with fusion of the right and left cusps and heavy cusp calcification extending to the annulus (*Figure 3*). A bioprosthetic valve was implanted in the supra-annular position using a non-everting mattress suture, with careful removal of the calcification adhering to the native valve. Notably, his ECG shortly after withdrawal from extracorporeal circulation showed complete restoration of AV conduction during temporary atrial pacing (*Figure 1C*). No recurrence of AVB was observed during the post-operative period (*Figure 1D*), and he was discharged to home after rehabilitation on post-operative Day 13. We have carefully monitored his outpatient follow-up with 24-h ECG, and no recurrence of AVB was observed up to 1 year post-operatively.

## Discussion

One serious complication of both surgical and transcatheter AVR is AVB, with reported frequencies of ∼5% for surgical and 10% for transcatheter AVR.^3^ The present case showed an unusual clinical course, diametrically opposed to what would normally be predicted, i.e. pre-operative persistent complete AVB that converted to normal sinus rhythm post-operatively. To the best of our knowledge, there has been only one case report analogous to this case in 1974.^4^

A recent cohort study reported that the major diagnosed cause of syncope in patients with severe AS was AVB rather than exercise-induced disproportionate cardiac output.^5^ Mechanisms of AVB in patients with AS have been reported for a century, and the primary cause is direct interference of the His bundle by calcification extending from the valvular annulus to the membranous septum.^6,7^ An additional driver has also been proposed: excessive strain stress on the septum by increased intraventricular pressure in the left ventricle.^4,8^ Intriguingly, the aetiology of AS in this patient was a bicuspid valve, as in the previous report,^4^ and bulky calcification was present on the leaflet and annulus of the valve that also extended towards the septum. One previous report noted that bacterial endocarditis in a patient with a bicuspid aortic valve generated a fistula beneath the right cusp, and the patient developed complete AVB.^9^ On Day 6 after AVR, that patient recovered to first-degree AVB. Although the same clinical course was suspected in the present case, the clinical history and pathological findings of the excised valve did not suggest the involvement of inflammatory conditions in the development of AVB. Collectively, one possible explanation for the restoration of conduction is that cautious surgical removal of the calcification extending into the septum and unloading of the ventricular pressure resulted in significant improvement in the function of AV conduction.

However, surgical elimination of a hindrance to the conduction system *per se* cannot fully explain the rarity of this patient’s clinical course. He had invariably experienced recurrent episodes of syncope following alcohol ingestion. This type of syncope is considered neurally mediated in many cases: a reduced compensatory response to alcohol-induced vasodilation and an imbalance between subsequent sympathetic activation and antagonistic parasympathetic activation.^10^ In terms of the relationship between alcohol and cardiac conduction disturbance, a case series study of alcohol-induced AVB reported that ethanol was suspected to cause an abnormal increase of vagal tone due to accentuated antagonism to sympathetic activity, which may further contribute to worsening of conduction slowing.^11^ The present patient is speculated to have similar dysregulation of the autonomic nervous system, which probably affected his AV conduction. We consider that increased vagal activity via the left ventricular mechanoreceptor evoked by intraventricular pressure overload due to the AS^12^ further exaggerated suppression of the conduction system. A marked improvement in cardiac pressure overload resulting from the release of AS may lead to the correction of the regulatory function of the autonomic nervous system and partly explain the normalization of AV conduction. Considering the significant involvement of the autonomic dysfunction, the narrow QRS of the escape beat during the complete AVB, and the reversibility of AVB, we infer that the primary level of AV conduction disturbance in the present case was at the atrio-nodal conduction tract.

To our best knowledge, this is the second case reported within half a century to show recovery from AVB after surgical replacement of the aortic valve. This extremely rare phenomenon cannot be explained by a simple or single mechanism. Multifactorial mechanisms, including haemodynamic impairment, mechanical compression by valvular calcification, autonomic imbalance, and hypersensitivity of the stretch-induced mechanoreceptor are considered based on our detailed assessments of the patient’s clinical course.

## Conclusions

We experienced a patient with an impressive clinical course of complete recovery from AVB following surgical AVR. The patient was planned for permanent pacemaker implantation post-operatively, but after the AVR, recovery from paradoxical AV conduction occurred. It is likely plausible that the amelioration of autonomic imbalance significantly contributed to the patient’s rare course, which cannot be explained solely by resolving mechanical problems in the cardiac conduction system. The present case provides new mechanistic insight into conduction system impairment in patients with AS.

## Lead author biography


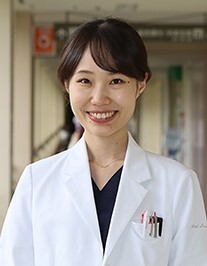


Dr Aya Nishihara graduated from Jichi Medical School in 2022. She completed her junior residency programme at Ibaraki Prefectural Central Hospital and is currently working in the Department of Dermatology at the University of Tsukuba Hospital.

## Supplementary Material

ytae549_Supplementary_Data

## Data Availability

The data underlying this article will be shared on reasonable request to the corresponding author.

## Notes

**Consent:** The authors confirm that written consent for the submission and publication of this case report was obtained from the patient in line with the COPE guidance.

**Funding:** This research received no specific grant from funding agencies in the public, commercial, or not-for-profit sectors.
